# Performance of Survivin mRNA as a Biomarker for Bladder Cancer in the Prospective Study UroScreen

**DOI:** 10.1371/journal.pone.0035363

**Published:** 2012-04-16

**Authors:** Georg Johnen, Katarzyna Gawrych, Heike Bontrup, Beate Pesch, Dirk Taeger, Séverine Banek, Matthias Kluckert, Harald Wellhäußer, Friedhelm Eberle, Michael Nasterlack, Gabriele Leng, Arnulf Stenzl, Thomas Brüning, and the UroScreen Study Group

**Affiliations:** 1 Institute for Prevention and Occupational Medicine of the German Social Accident Insurance, Institute of the Ruhr University Bochum (IPA), Bochum, Germany; 2 Institute of Urology, Eberhard Karls University, Tübingen, Germany; 3 German Social Accident Insurance's Institution for the Raw Materials and Chemical Industry (BG RCI), Heidelberg, Germany; 4 Occupational Medicine and Health Protection, BASF SE, Ludwigshafen, Germany; 5 Safety - Health Protection, Currenta GmbH & Co. OHG (formerly Bayer), Leverkusen, Germany; The University of Texas M. D. Anderson Cancer Center, United States of America

## Abstract

**Background:**

Urinary biomarkers have the potential to improve the early detection of bladder cancer. Most of the various known markers, however, have only been evaluated in studies with cross-sectional design. For proper validation a longitudinal design would be preferable. We used the prospective study UroScreen to evaluate survivin, a potential biomarker that has multiple functions in carcinogenesis.

**Methods/Results:**

Survivin was analyzed in 5,716 urine samples from 1,540 chemical workers previously exposed to aromatic amines. The workers participated in a surveillance program with yearly examinations between 2003 and 2010. RNA was extracted from urinary cells and survivin was determined by Real-Time PCR. During the study, 19 bladder tumors were detected. Multivariate generalized estimation equation (GEE) models showed that β-actin, representing RNA yield and quality, had the strongest influence on survivin positivity. Inflammation, hematuria and smoking did not confound the results. Survivin had a sensitivity of 21.1% for all and 36.4% for high-grade tumors. Specificity was 97.5%, the positive predictive value (PPV) 9.5%, and the negative predictive value (NPV) 99.0%.

**Conclusions:**

In this prospective and so far largest study on survivin, the marker showed a good NPV and specificity but a low PPV and sensitivity. This was partly due to the low number of cases, which limits the validity of the results. Compliance, urine quality, problems with the assay, and mRNA stability influenced the performance of survivin. However, most issues could be addressed with a more reliable assay in the future. One important finding is that survivin was not influenced by confounders like inflammation and exhibited a relatively low number of false-positives. Therefore, despite the low sensitivity, survivin may still be considered as a component of a multimarker panel.

## Introduction

Bladder cancer is one of the leading cancers in the U.S. and in Europe. In Germany, the incidence is about 29,000 cases per year [1]. Because of the high rate of tumor recurrence, close monitoring and repeated use of therapies are necessary. As a consequence, bladder cancer is the most costly cancer disease [2]. Tumors of the urinary bladder can be caused by occupational exposure to aromatic amines but tobacco smoking is considered to be the strongest contributor to the development of these malignancies [3].

The availability of effective treatments, the access to the target organ via urine, and the relatively good overall survival makes bladder cancer a candidate for screening programs in high-risk populations [4]. Unfortunately, cystoscopy, which is the current gold standard for bladder cancer detection, is an invasive and rather painful method. In some countries high costs may also play an important role [4]. These facts preclude cystoscopy from being used in screening cohorts. In contrast, urinary tumor markers are non-invasive tools to detect bladder cancer. Typical markers are proteins, RNA, DNA, metabolites, or cellular features, which have the advantage that they can be determined in urine samples [5], [6]. Of the numerous known markers, however, only few have been tested in prospective studies or trials and were successful to be approved by the FDA. Consequently, more longitudinal studies are necessary to prove the value of these markers for cancer screening and clinical decision-making [7].

Survivin is a relatively small protein of 16.4 kDa encoded by the gene *BIRC5* [8], [9]. Its three-dimensional structure was resolved and suggested an adaptor or docking function [10], [11]. Binding to a number of other macromolecules has in fact been demonstrated and survivin emerged as a central node in multiple cellular networks [9], [12]–[14]. While survivin belongs to the inhibitor of apoptosis (IAP) gene family its functions are not restricted to a regulatory role in apoptosis. Other functions are the control of cell division and chromosome segregation, promotion of proliferation, stress response and angiogenesis, and it plays a role in metastasis [9], [15]–[19]. Dysregulation of survivin would therefore affect four of the six so-called ‘hallmarks of cancer’ in the model of tumorigenesis described by Hanahan and Weinberg, suggesting a central function of survivin in carcinogenesis and tumor progression [20].

The multiple mechanistic roles of survivin are also reflected in its widespread occurrence in all stages of tumor development, though with preference to later stages in several cancers [21]–[25]. Survivin is overexpressed in most human cancers but rarely detectable in healthy adult tissues [8], [26]. It has, therefore, been proposed as a potential tumor marker and target for therapy [8], [27]. Due to the fact that non-invasive detection in urine samples is possible, survivin could be used specifically for screening of urogenital malignancies. Several studies, mostly of cross-sectional design and with a relatively limited number of cases, have demonstrated that survivin is a promising candidate for further validation in longitudinal studies [28]–[34]. Shariat *et al.* performed a larger prospective study on recurrent bladder cancer that was based on immunohistochemical staining of tumor samples. Herein, survivin improved the prediction of recurrence and survival in a subgroup of patients [21].

In the present study, we determined survivin in the prospective screening cohort UroScreen [35]–[37] using an mRNA-based assay in order to validate its function as a tumor marker for early detection of bladder cancer.

## Results

**Figure 1:**
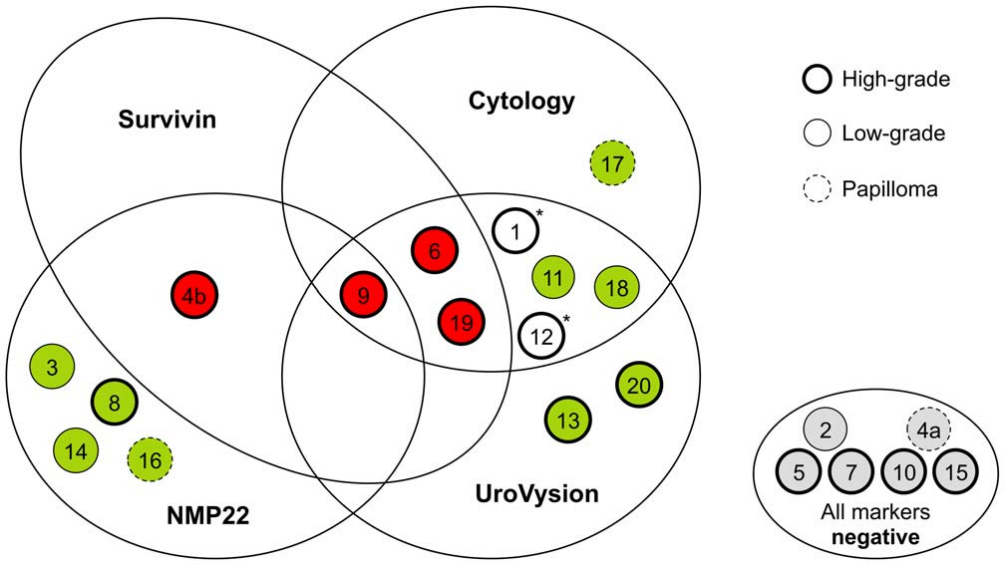
Venn diagram of all cases detected in UroScreen and correlation with marker results. A total of 19 tumors were detected in 18 cases. Survivin, cytology, NMP22, and UroVysion detected 13 of the tumors. *For two additional tumors (number 1 and 12) no sample was available for survivin determination.

The participants of the UroScreen cohort were active or retired chemical workers with former exposure to aromatic amines as described previously [35]–[37]. They were examined between September 2003 and June 2010. Urine samples were collected for urine status, cytology, the determination of NMP22®, chromosomal aberrations (UroVysion™), and – if sufficient material was available – for survivin [36], [37]. For survivin measurements 5,716 urine samples could be obtained from 1,540 participants (Tables 1 and 2). Median age of the cohort was 62 years (range 27–90 years). Of the 1,540 persons, 18 developed tumors, including one person who developed two tumors during the study resulting in 19 tumors in total for the investigation of survivin. For another two cases no suitable urine sample was available for survivin determination. Of the 19 tumors, three were papillomas, eleven high-grade, and five low-grade tumors (Table 3). Cytology detected eight tumors. The survivin assay detected four tumors, all of which were high-grade. Three of those were also detected by cytology and other markers; one was detected by survivin and NMP22 only (Figure 1).

**Table 1:** Characteristics of male participants of UroScreen with former occupational exposure to aromatic amines.

Characteristics	Category	All	Cases	Non-cases
Subjects		1540	18* (1.2%)	1522 (98.8%)
Age in 2010 (years)	Median (range)	62 (27–90)	68 (43–80)	62 (27–90)
	Age at diagnosis		66 (38–76)	
Smoking status at baseline	Never	424	14	420
	Ever	1116	4	1102
Former bladder cancer at baseline		18	2	16

* One person with two tumors.

**Table 2:** Characteristics of urine samples of the UroScreen study.

Characteristics	Category	All	Cases	Non-cases	
Number of urine samples		5716 (100%)	75 (1.3%)	5641 (98.7%)	
				Test result for survivin	
				True negative	False positive
				5435	206
Creatinine*	N	4806	63	4552	191
	Median	1.15	1.01	1.15	1.19
	(Inter-quartile range)	(0.68–1.64)	(0.57–1.53)	(0.68–1.64)	(0.76–1.56)
β-actin	Median	16600	24600	15900	73600
	(Inter-quartile range)	(5770–48950)	(9850–77600)	(5540–45700)	(20880–221000)

* Creatinine was only available for the 4806 samples of subcohort A.

**Table 3:** Characteristics and test results of cases.

Case^1^	Histopathological finding	Months before diagnosis	Creatinine (g/l)	Leukocytes^2^	Albumin (mg/l)	β-actin (copies)	Survivin (copies)	Assay^3^	Test result survivin	Test result NMP22 [37]	Test result cytology
2	Low-grade	10	0.23	Traces	Missing	872000	82.8	Qiagen/FDI	Negative	Negative	Negative
3	Low-grade	2	1.59	Traces	Missing	1020	48	Qiagen/FDI	Negative	Positive	Negative
4a	Papilloma	10	0.35	Traces	Missing	73500	51.5	Qiagen/FDI	Negative	Negative	Negative
4b	High-grade	24	0.87	Traces	1556	363000	83000	Invitek/FDI	Positive	Positive	Negative
5	High-grade	26	1.01	Traces	Missing	125000	61.9	Qiagen/FDI	Negative	Negative	Negative
6	High-grade	1	0.24	None	2	80800	88200	Invitek/FDI	Positive	Negative	Positive
7	High-grade	14	2.92	Traces	19	136000	5870	Invitek/FDI	Negative	Negative	Negative
8	High-grade	0/14	1.53	Medium	82	2260000	1300	Qiagen/FDI	Negative	Positive	Negative
9	High-grade	2	0.32	Medium	63	295000	52400	Invitek/FDI	Positive	Positive	Positive
10	High-grade	18	2.93	Traces	22	2370	3820	Invitek/FDI	Negative	Negative	Negative
11	Low-grade	2	0.44	None	22	2040	1	Invitek/FDI	Negative	Negative	Positive
13	High-grade	1	Missing	Missing	Missing	74300	4580	Invitek/FDI	Negative	Negative	Negative
14	Low-grade	3	0.74	Traces	35	25200	85900	Invitek/IPA	Negative	Positive	Negative
15	High-grade	2	0.72	Traces	13	11400	42.3	Invitek/FDI	Negative	Negative	Negative
16	Papilloma	2	Missing	Missing	Missing	11300	19.5	Invitek/IPA	Negative	Positive	Negative
17	Papilloma	2	0.77	Traces	2	81200	12000	Invitek/IPA	Negative	Negative	Positive
18	Low-grade	2	1.06	Missing	22.2	45100	50600	Invitek/IPA	Negative	Negative	Positive
19	High-grade	3	Missing	Missing	Missing	280000	229000	Invitek/IPA	Positive	Negative	Positive
20	High-grade	6	0.44	Missing	8.7	9930	10	Invitek/IPA	Negative	Negative	Negative

Results are from the last screening round before diagnosis.

1 Cases number 1 and 12 are omitted because no samples were available for survivin determination.

2 Traces = 1–5 leukocytes, medium = 5–<250 leukocytes, abundant ≥250 leukocytes (dipstick or microscopic sediment analysis).

3 cut-off: 10,000 (Qiagen/FDI), 40,000 (Invitek/FDI), 100,000 (Invitek/IPA).

The tumor marker survivin was determined by an mRNA-based assay that was not commercially available. During the seven years of this longitudinal study, unforeseen events regarding the reliability and availability of assay components (RNA isolation kits from Qiagen and Invitek, survivin reagents from FDI and IPA) prompted us to modify the assay design twice, leading to three different assay variants (Qiagen/FDI, Invitek/FDI, Invitek/IPA). We standardized the survivin copy numbers and could demonstrate by using a multivariate generalized estimation equation (GEE) model that the assay variants did not confound the test results when implementing the standardized survivin copy numbers (Table 4). All other results were, therefore, derived from the standardized values.

**Table 4:** Potential predictors of a positive survivin test result based on GEE models that included only urine samples with complete information on all variables (190 positive survivin tests in 4546 samples from 1273 participants of UroScreen).

Variable at sampling	Category	N (N_pos_)	OR	95% CI
Leukocytes	None	1369 (50)	1	
	Traces	2884 (110)	0.82	0.57–1.17
	Non-abundant and abundant	293 (30)	0.80	0.47–1.39
Hematuria	None or traces	3584 (140)	1	
	Microhematuria and gross hematuria	962 (50)	1.08	0.76–1.53
Creatinine	<0.5 g/l	733 (30)	1.29	0.84–1.99
	0.5–2.5 g/l	3567 (151)	1	
	>2.5 g/l	246 (9)	0.76	0.39–1.50
Log_10_ (β-actin)		4546 (190)	**3.11**	**2.51–3.85**
Age in 10 years		4546 (190)	1.09	0.95–1.25
Smoking status	Never	1302 (52)	1	
	Ever	3244 (138)	11.04	0.75–1.45
Prevalent bladder cancer	None	4492 (189)	1	
	Yes	54 (1)	0.21	0.03–1.46
Bladder cancer*	None	4506 (186)	1	
	Yes	40 (4)	**2.54**	**1.02–6.33**
Assay**	Qiagen/FDI	876 (28)	1	
	Invitek/FDI	1947 (87)	1.17	0.75–1.84
	Invitek/IPA	1723 (75)	1.01	0.64–1.58

This analysis was performed with samples of subcohort A only because not all parameters were available for the full data set. N_pos_: number of samples positive for survivin, OR: odds ratio, CI: confidence interval.

* Bladder cancer detected during UroScreen.

** After standardization.

Potential predictors of a positive survivin result were explored as shown in Table 4. Indicators of infection and inflammation (leukocytes), hematuria, or factors like age, smoking, or previous bladder cancer did not show a significant effect on survivin levels. The ‘concentration’ (specific density) of the urine sample, reflected in the urinary creatinine concentration, tended towards an inverse correlation with survivin, but this influence was not significant. As expected, bladder cancer observed during UroScreen was a predictor of positive survivin tests (OR 2.54, 95% CI 1.02–6.33). β-actin (OR 3.11, 95% CI 2.51–3.85) was an effect modifier, indicating a strong influence of the amount and quality of the recovered mRNA on the PCR performance. A tenfold increase in the copy numbers of β-actin was associated with a threefold higher probability of a positive survivin test. We tested whether β-actin was correlated with urine density by calculating the Spearman's rank correlation coefficient. There was a significant but only weak association of β-actin copy numbers with urinary creatinine as a proxy of urine density (r_s_ 0.17, 95% CI 0.12–0.23).

The cancer-predictive values for survivin are listed in Table 5. Calculations were based on the test results obtained in urine samples from the last screening round before diagnosis. Survivin reached a sensitivity of 21.1% for all tumor entities, 25.0% for all tumors but without papillomas, and 36.4% for high-grade tumors only. It did not detect any of the five low-grade tumors. The specificity was 97.5% in all (sub)groups (all tumors, tumors without papillomas, high-grade, and low-grade tumors). The positive predictive value (PPV) reached 9.5% in all groups, whereas the negative predictive value (NPV) ranged between 99.0 and 99.7%.

**Table 5:** Cancer predictive values of the last survivin test before diagnosis of bladder cancer.

Result	All tumors	Tumors without papillomas	High-grade	Low-grade
	N = 19	N = 16	N = 11	N = 5
True positive	4	4	4	0
False negative	15	12	7	5
True negative	1484	1484	1484	1484
False positive	38	38	38	38
Sensitivity	21.05	25.00	36.36	-
Specificity	97.50	97.50	97.50	97.50
Positive predictive value	9.52	9.52	9.52	-
Negative predictive value	99.00	99.20	99.53	99.66

**Figure 2:**
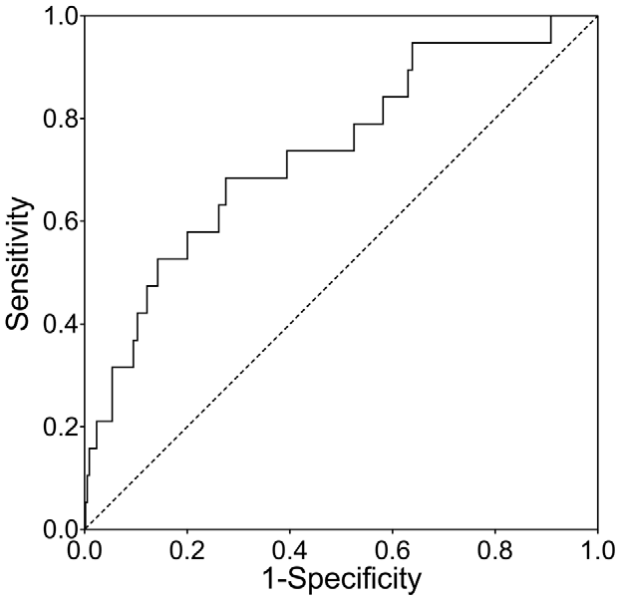
ROC curve for log_10_ (survivin) in the last screening round before diagnosis, adjusted for log_10_ (β-actin) and age in 10-year classes. Analysis was performed for all tumors entities. The resulting area under curve (AUC) was 0.74 with a 95% CI (confidence interval) of 0.61–0.86.

Receiver operating characteristic (ROC) analyses were performed with standardized survivin levels from the last screening round before diagnosis and adjusted for log_10_ (β-actin) and age in 10-year classes. They resulted in area under curve (AUC) values of 0.74 (95% CI 0.61–0.86) for all tumor entities (Figure 2), 0.75 (95% CI 0.60–0.89) for all tumors without papillomas, 0.80 (95% CI 0.66–0.94) for high-grade tumors, and 0.66 (95% CI 0.36–0.96) for low-grade tumors only. To judge the performance of the assay variants, we calculated adjusted ROC curves for each variant. The resulting AUCs were 0.70 (95% CI 0.34–1.00) for Qiagen/FDI, 0.80 (95% CI 0.54–1.00) for Invitek/FDI, and 0.79 (95% CI 0.67–0.91) for the assay variant Invitek/IPA.

Possible reasons for negative test results in cases are compiled in Table 6. The last samples of cases 5 and 10 were obtained 26 and 18 months before tumor diagnosis, respectively. Case 8 showed a positive test result 14 months before diagnosis but the sample shortly before diagnosis was negative. All other false-negative results were in cases with papillomas and low-grade tumors; 31% were associated with a low density of the collected urine indicated by low creatinine (<0.50 g/l), and another 20% were likewise associated with low RNA integrity as indicated by β-actin levels <2,500 copies. In comparison, 15% and 11% of all true-negative samples in the last screening round were associated with low creatinine and low β-actin, respectively.

**Table 6:** Selected sample characteristics of false-negative cases.

Case number^1^	Months before diagnosis	Histopathology	Quality of urine	Quality of RNA
2		Low-grade	Creatinine low	
3		Low-grade		β-actin low
4a		Papilloma	Creatinine low	
5	26			
7				
8	0/14^2^			
10	18			β-actin low
11		Low-grade	Creatinine low	β-actin low
13				
14		Low-grade		
15				
16		Papilloma		
17		Papilloma		
18		Low-grade		
20			Creatinine low	

1 For cases 1 and 12 no samples were available for survivin determination.

2 Negative at last (0 months), positive at previous screen (14 months).

## Discussion

While numerous potential markers have been described for the early detection of bladder cancer, none so far have been implemented in clinical guidelines for screening or clinical follow-up on recurrence. Ideally, marker assays suitable for clinical practice should be robust and cheap as well as fast and in an easy-to-use format, e.g., a point-of-care test [38]. However, before the assay format can be optimized, the performance of the marker itself has to be evaluated. For molecular markers like survivin the development of stable and simple assays can be promising if their performance with currently available assays in bladder cancer screening has been demonstrated to be comparable with or better than approved tests. In UroScreen, we determined survivin with a relatively complex assay wherein positive test results were not followed-up with cystoscopy. Overall, survivin was detected in particular in high-grade bladder cancer and its performance was comparable to the other markers tested. However, mRNA integrity was an important modifier of positive test results, and therefore a more robust test should be developed.

To prove the value of new markers for clinical decision-making, their performance has to be assessed in longitudinal studies and clinical trials [7]. UroScreen was a prospective cohort study with more than 1,500 chemical workers aimed to assess NMP22, UroVysion, and survivin as tumor markers [36], [37]. It was the first prospective study that investigated the performance of survivin in the early detection of bladder cancer. For 19 of the bladder tumors that were detected during the conduct of the study sufficient sample material was left for survivin determination. The low incidence of bladder cancer in UroScreen as well as in the general population is a critical issue for bladder cancer screening [4]. UroScreen was established as an extension of an already established surveillance program in chemical workers [35]. The initial power calculations were based on former data showing at that time a higher tumor incidence in the study population [39]. Currently, the incidence in this original high-risk cohort has reached a level closer to that of the general population due to the ban of carcinogenic aromatic amines from use in the chemical industry decades ago. It is further likely that technological progress and measures to improve work safety contributed to lowering the bladder cancer incidence in this cohort of workers from two large chemical companies. In addition, the median age of the UroScreen cohort in 2010 was 62 years, whereas the median age for bladder cancer for men in Germany was 72 years [1].

It has been shown before that survivin expression is higher and more frequent in high-grade tumors or later stages of cancer development [21], [23]–[25]. In accordance with this observation, survivin did not detect cases with papilloma or low-grade bladder cancer in UroScreen, whereas the sensitivity was better for high-grade tumors. If this could be confirmed, survivin might be a useful adjunct for the follow-up of patients with faster growing tumors where detection should be as early as possible. Here, a non-invasive marker panel would be a promising approach to detect recurrence sooner and reduce the number of cystoscopies [40]. Nevertheless, individual molecular markers are currently lacking sufficient sensitivity to replace cystoscopy [38].

The low number of incident cases limited the power of the study to assess the performance of the tumor markers and contributed to their low PPVs. Previously published studies showed specificities between 88% and 100% and sensitivities between 53% and 100% [28]–[30], [32]–[34]. While our results showed a similar specificity (98%) the sensitivity (21%) was markedly lower in this cohort study. In part, this may be due to the different assays applied. The main reason for the differences most likely is, however, the cross-sectional study design of the other studies [41]. Major shortcomings are the lack of consideration of the dimension time in order to calculate predictive values, and a potential selection bias because cases and controls have been recruited from different populations. The longitudinal and thus prospective design of the UroScreen study avoids this bias. It also represents a setting that is closer to clinical practice.

We observed various reasons that might be responsible for the fact that cases were not detected in our cohort study. A lack of compliance in a voluntary screening study is a typical problem that influences the early detection of cases. For most of the participants of the study, samples were not available for each consecutive year. In several cases, the difference between sample acquisition and tumor diagnosis was more than twelve months, in one case even 26 months. It can be expected that an increase of marker levels is less likely with an increasing time interval between sample collection and diagnosis. Another problem was that participants frequently voided urine shortly before they arrived for an appointment and therefore the urine collected on-site was insufficient in quality and/or quantity for some of the marker tests. This kind of urine was typically associated with low creatinine, a low number of sedimented cells, and low β-actin.

In terms of confounding, survivin was less influenced than NMP22 and UroVysion by urine status [36], [37]. According to the GEE model, creatinine and leukocytes did not influence the test results for survivin. As bladder infections are frequently observed in the elderly, it is important that inflammatory processes do not influence a tumor test. This is an advantage the survivin assay has in particular over NMP22, which is known to be frequently false positive when participants suffer from infections. It was reflected in the lower number of false-positive results of the survivin assay compared to the NMP22 assay [37]. Despite the limited sensitivity the relatively high specificity might allow survivin to be added to a marker panel. It is important to note that positive survivin tests did not result in a recommendation for cystoscopy. As a result, there is a possibility that a few tumors might have remained undetected and that we underestimated the performance of survivin. This impairs the comparison with the performance of the FDA-approved tests, i.e. NMP22 and UroVysion. The survivin assay was positive for one case (4b) that was not detected by cytology or UroVysion. Survivin was also positive for case number 8, which was not detected by the cell-based assays 14 months before diagnosis. Both cases were additionally tested positively by NMP22. However, more cases would be required to prove the point that expression-based markers like survivin might complement the cell-based assays UroVysion and cytology.

A specific issue with the quantification of survivin was the difficulty to maintain the quality of a still experimental assay over a period of several years. This can be a design-specific problem of prospective cohort studies in comparison to cross-sectional studies. Problems due to changes of the original RNA isolation kit and the discontinuation of the production of assay reagents by the original supplier resulted in three assay variants that led to different cut-off values for survivin positivity. The discontinuities prevented the determination of an optimized overall cut-off for the complete data set. We normalized the copy numbers and implemented the assay into the GEE model as potential confounder in order to test if residual confounding could be found with the standardized survivin data. The assay did not influence the test results but β-actin turned out to be a significant modifier of the test results. It appears possible that the specificity of survivin was reduced if more of the urine samples had been higher concentrated. However, the concentration of β-actin was not simply a function of urine density because the correlation with creatinine was weak, indicating that other factors like mRNA integrity may have contributed to the performance of β-actin in the PCR reactions.

Besides these unforeseen methodological factors that influenced the assay, the widely used mRNA format to detect survivin also contributed to the overall performance of the assay. The RNA integrity assessed by copies of β-actin was evaluated as the strongest influence on the test results. The rationale behind the use of an mRNA-based assay for survivin was to be able to detect even weak signals of survivin that are present in the small numbers of exfoliated urothelial cells. PCR and RT-PCR are elegant and well-established methods to amplify and quantify very small amounts of nucleic acids. Nonetheless, the low stability of mRNA in general, even with the addition of RNase inhibitors, limits the applicability of such assays for samples collected outside the controlled conditions of a laboratory setting. There are better RNase inhibitors available nowadays that would even dispense with the necessity of sample freezing [42]. But the addition of these preservatives to the sample has to be immediate. A possible way to avoid delays would be a special collection tube, similar to those used for blood collection, which already contains the preservative. Another problem inherent to the survivin assay we used was the nonlinearity caused by the two PCR-based amplification steps that limited the accuracy of quantification and strategies for normalization. Even applying β-actin, which served as an internal control, for correction was not always sufficient to compensate for the large variations in mRNA content in the samples, probably because of the additional preamplification step of the survivin assay. For that reason, for clinical settings under real life conditions, more stable molecules like proteins might be better targets. While a PCR-like amplification of proteins is not possible, ELISA-based assays can achieve very good sensitivities and are now available for survivin in better quality than at the onset of the UroScreen study.

To date, UroScreen is the largest prospective study to evaluate survivin for the early detection of bladder cancer in a cohort of asymptomatic participants. Despite the low number of incident cases, valuable information has been gained on the performance of the mRNA-based assay of survivin, technical challenges, influence of confounders, and cancer predictive values. A more robust assay would greatly benefit the marker and its use in clinical practice. Survivin may have the potential to improve detection, especially of high-grade tumors; its sensitivity, however, rests on only four tumors (of 19) that were detected with three different assay variants. The high specificity implies that survivin might be considered to serve as part of a multimarker panel to complement other markers but further validation in a prospective study with more cases is warranted. Testing survivin within a cohort of patients with a high risk of recurrent tumors would be a promising approach.

## Materials and Methods

### Study population and diagnosis of tumors

Participants were recruited from the ODIN (Organisationsdienst für nachgehende Untersuchungen) cohort within the frame of a surveillance program of the statutory accident insurance of the chemical industry that offers yearly examinations of active and retired workers who have been exposed to aromatic amines. From September 2003 to June 2010 1,609 male workers at two large chemical sites in Germany (subcohort A: BASF, Ludwigshafen; subcohort B: Bayer, Leverkusen) participated in the UroScreen study. 1,540 of those provided sufficient sample material for survivin measurements. A questionnaire was applied to document smoking habits and relevant diseases. All participants gave written informed consent. The study was approved by the ethics committee of the University of Tübingen (No. 1/2003V).

As the other tumor tests (cytology, NMP22®, UroVysion™) applied in UroScreen were approved in contrast to survivin, it had lower priority when sample material was limited. Therefore, only 5716 of the 7091 urine samples were available for survivin measurements. Characteristics of the cohort are summarized in Tables 1–2 and are described in more detail elsewhere (Pesch et al., submitted and [37]). Positive test results for cytology, NMP22®, or UroVysion™ resulted in a recommendation for a cystoscopic examination, while positive results for the non-approved survivin assay did not.

As of November 2011, 21 tumors in 20 persons were detected by cystoscopy and confirmed by reference pathology. For 18 of the cases (19 tumors) urine samples were available for survivin determination. One case had two tumors (4a and 4b in Table 3). Histopathological findings of all 21 tumors and parameters of the corresponding urine samples (last screening round before diagnosis of a tumor) are depicted in Table 3 and in [37].

### Urine collection

Urine samples were collected on site at the chemical plants in Ludwigshafen and Leverkusen. For survivin determination, samples of 40–50 ml spontaneously voided urine were centrifuged in a swinging bucket rotor at 500× g for 10 minutes at 10°C. Supernatant was carefully removed and cell pellets were mixed with 500 µl Lysis Solution R (Invitek, Berlin, Germany), which contains RNase inhibitors. Finally, the cell sample was frozen at −20°C and sent to the laboratory in Bochum where they were stored until RNA isolation. Handling of samples for determination of urine status and other markers is described below.

### Urine status and assessment of hematuria

In all samples the urine status was determined in fresh urine (before centrifugation) as described previously [36]. Urinary creatinine was determined with the CREA plus® test (Roche Diagnostics, Mannheim, Germany). Erythrocytes, hemoglobin (Hb), leukocytes, albumin and other parameters were determined with Combur 10 test® strips (Roche). In addition, erythrocytes and leukocytes were also determined semi-quantitatively in urine sediment. Creatinine measurements were available for subcohort A only (4806 samples). For 4546 samples of these, complete information on all parameters (erythrocytes, leucocytes, etc.) was available.

### NMP22®, UroVysion™, and cytology

Nuclear matrix protein 22 (NMP22) was determined quantitatively with the NMP22® ELISA kit (Matritech/Alere GmbH, Köln, Germany) according to the manufacturer's protocol. The cut-off for positive results was set to 10 units/ml. Chromosomal instability in sedimented urothelial cells was assessed using the UroVysion™ Bladder Cancer Kit (Abbott Laboratories, Abbott Park, IL). The test was considered positive if at least four nuclei had three signals of two or three chromosomes (3, 7, and 17) or at least 12 nuclei showed a signal for the 9p21 locus. Urinary cytology was performed as described previously [36], [43].

### RNA isolation and survivin assays

Quantification of survivin was based on Real-Time PCR with mRNA isolated from exfoliated urothelial cells in urine. For the determination of survivin three variants of the mRNA-based assay were employed. The variations were a consequence of changes by the manufacturers of the RNA isolation kit and the RT-PCR reagents, respectively. Initially, the protocol and reagents for the survivin assay were provided by Fujirebio Diagnostics, Inc. (FDI, Malvern, PA). The FDI protocol recommended the RNeasy Mini Kit (Qiagen, Hilden, Germany) for RNA isolation. This kit, however, is not optimized for the very small amount of RNA that is usually retrieved from the low number of cells obtained from 40–50 ml of urine, leading to mostly low yields. A slight change in one of the components of the kit in 2005 led to a further drop in RNA yield. For that reason, it was replaced by an alternative isolation kit (InviTrap® Spin Cell RNA Mini Kit) that produced better and more consistent yields.

In 2007, FDI discontinued the production of reagents for the Real-Time PCR assay. This prompted another change in the assay procedure in 2008. Because no other source was available, we had to design and produce some of the assay components ourselves (in the following called “IPA assay”). Slight differences in the primers and probes of the Real-Time PCR assay consistently led to higher copy numbers of survivin compared to the original FDI assay. The three assay variants are as follows:

### Variant 1 (Qiagen kit/FDI assay)

This variant was used from September 2003 until October 2005. RNA isolation was performed with RNeasy Mini Kits (Qiagen) according to the manufacturer's protocol. Reverse transcription of mRNA, preamplification, and quantitative Real-Time PCR were done as described by Kenney et al., except that the reaction volumes of each step were cut in half to 25 µl [33]. Primers, reagents, and positive controls were provided by FDI at no charge. In contrast to the published assay, instead of an ABI PRISM Sequence Detection System a LightCycler II system (Roche) was used. To be able to use the capillaries of the LightCycler system it was necessary to add bovine serum albumin (BSA, AppliChem, Darmstadt, Germany) to the TaqMan reaction, with a final concentration of 0.16 µg/µl. The cut-off for survivin-positive samples was set to 10,000 copies of survivin mRNA as recommended by FDI. Parallel quantification of β-actin, without the preamplification step, served as a quality control. Samples with less than 1,000 copies of β-actin were excluded.

### Variant 2 (Invitek kit/FDI assay)

This variant was employed from October 2005 until March 2008 and was identical to variant 1, except for the RNA isolation step. Here, instead of the RNeasy kit, an InviTrap® Spin Cell RNA Mini Kit (Invitek) was used according to the manufacturer's protocol. The cut-off for survivin-positive samples was set to 40,000 copies of survivin mRNA.

### Variant 3 (Invitek kit/IPA assay)

This variant was used from March 2008 until the end of the study in 2010. RNA was isolated using the InviTrap® Spin Cell RNA Mini Kit (Invitek), as in variant 2. The assay (IPA assay) to quantify survivin and β-actin was very similar to the original FDI assay used in variant 1 and 2, except for small differences in some of the primers and probes [33]. Details of the assay are described below. The cut-off for survivin-positive samples was raised to 100,000 copies of survivin mRNA.

### Reverse transcription

In all assay variants cDNA was synthesized using a TaqMan® Reverse Transcription Reagent kit (Applied Biosystems, Foster City, CA). The 25 µl reaction volume contained 2.5 µl 10× RT buffer, 5 µl MgCl_2_, 1.75 µl dNTP mixture, 0.75 µl random hexamers, 1 µl oligo (dT)_16_, 1 µl RNase inhibitor, 1 µl reverse transcriptase, and 12 µl RNA sample (or control). The reaction was incubated at 42°C for 80 min and then heat inactivated at 95°C for 5 min.

### Preamplification

The low abundance of survivin in urine samples required a preamplification step. This step was not necessary for β-actin. The following primers were used to amplify the survivin cDNA: 5′-ATG GGT GCC CCG ACG TTG CC-3′ (forward) and 5′-GCT CCG GCC AGA GGC CTC AA-3′ (reverse). The primers were synthesized by Eurofins MWG Operon (Ebersberg, Germany) and provided in a concentration of 5 pmol/µl (HPSF-purified). The 20 µl PCR reaction contained 10 µl 2× TaqMan® Universal PCR Master Mix with UNG (uracil-N-glycosylase) (Applied Biosystems), 1 µl forward primer, 1 µl reverse primer, 4 µl DEPC-treated H_2_O (Roth, Karlsruhe, Germany), and 4 µl cDNA. The thermocycler was programmed as follows: 1 cycle 50°C for 2 min and 95°C for 10 min, 20 cycles 95°C for 30 sec, 55°C for 1 min, and 72°C for 1 min.

### Real-Time PCR

The preamplified cDNA of survivin and the cDNA of β-actin were quantified by Real-Time PCR. Both LightCycler-based measurements were carried out in parallel with an identical PCR protocol: 1 cycle of 50°C for 2 min and 95°C for 10 min followed by 45 cycles of 95°C for 15 sec and 60°C for 1 min. The primers and probes were synthesized by Applied Biosystems with the following sequences: 5′-GAT GAC GAC CCC ATA GAG GAA C-3′ (survivin forward, 10 pmol/µl), 5′-GGG TTA ATT CTT CAA ACT GCT TCT-3′ (survivin reverse, 10 pmol/µl), VIC-5′-TCC GGT TGC GCT TTC CTT TCT GTC-3′-TAMRA (survivin probe, 12 pmol/µl) and 5′-CCT GGC ACC CAG CAC AA-3′ (β-actin forward, 10 pmol/µl), 5′-GCC GAT CCA CAC GGA GTA CT-3′ (β-actin reverse, 10 pmol/µl), FAM-5′-AAG ATC AAG ATC ATT GCT CCT CCT GAG CG-3′-TAMRA (β-actin probe, 12 pmol/µl).

For the survivin quantification the 20 µl RT-PCR reaction contained 10 µl 2× TaqMan® Universal PCR Master Mix without UNG (Applied Biosystems), 2 µl survivin forward primer, 2 µl survivin reverse primer, 1 µl survivin probe, 1 µl H_2_O (Roth), 0.4 µl BSA (10 µg/µl, Roche), and 3.6 µl preamplified cDNA of survivin (or positive control, negative control, or standards of survivin). For β-actin quantification the 20 µl reaction contained 10 µl 2× TaqMan® Universal PCR Master Mix with UNG (Applied Biosystems), 2 µl β-actin forward primer, 2 µl β-actin reverse primer, 1 µl β-actin probe, 1 µl H_2_O (Roth), 0.4 µl BSA (10 µg/µl, Roche), and 3.6 µl cDNA of β-actin (or positive control, negative control, or standards of β-actin).

Recombinant survivin and β-actin cDNA were each subcloned into a pDrive vector (Qiagen) and served as positive controls: A dilution of about 10,000 copies of survivin mRNA (as well as a separate reaction with about 10,000 copies of β-actin mRNA) was run in parallel with each reverse transcription and the following steps.

For the standard curve, standard A contained 100, standard B 1,000, standard C 10,000, standard D 100,000, and standard E 1,000,000 copies of survivin/β-actin cDNA (each). DEPC-treated H_2_O (Roth) served as negative control.

### Statistical analysis

Survivin was quantified in three assay variants. The logarithm of survivin was standardized by subtracting the assay-specific mean and dividing by the assay-specific standard deviation. For the evaluation of the performance of survivin the last urine sample during follow-up and for cases the last sample before diagnosis was used. To evaluate potential predictors for a positive test result multivariate generalized estimation equation (GEE) models were applied. In these models the following parameters were included: age in 10-year classes, smoking status (never vs. ever), prevalent bladder cancer and bladder cancer observed during UroScreen, diabetes mellitus, and urine status (creatinine, hematuria, and leukocytes). Detailed data on urine status was available for a sub-cohort. Hematuria and leukocytes were semi-quantitatively assessed according to [36]. ROC curves and AUC values were determined for the full dataset and different subsets. Cancer predictive values were calculated for all tumors and subtypes with 95% confidence intervals (CI). All calculations were performed with SAS/STAT and SAS/IML software, version 9.2 (SAS Institute Inc., Cary, NC).

## References

[1] Koch-Institut Robert, Gesellschaft der epidemiologischen Krebsregister in Deutschland e.V. (2010). Krebs in Deutschland 2005/2006. Häufigkeiten und Trends.

[2] Lotan Y, Kamat AM, Porter MP, Robinson VL, Shore N (2009). Key concerns about the current state of bladder cancer: a position paper from the Bladder Cancer Think Tank, the Bladder Cancer Advocacy Network, and the Society of Urologic Oncology.. Cancer.

[3] Freedman ND, Silverman DT, Hollenbeck AR, Schatzkin A, Abnet CC (2011). Association between smoking and risk of bladder cancer among men and women.. JAMA.

[4] Lotan Y, Svatek RS, Sagalowsky AI (2006). Should we screen for bladder cancer in a high-risk population?: A cost per life-year saved analysis.. Cancer.

[5] Habuchi T, Marberger M, Droller MJ, Hemstreet GP, Grossman HB (2005). Prognostic markers for bladder cancer: International Consensus Panel on bladder tumor markers.. Urology.

[6] Lokeshwar VB, Habuchi T, Grossman HB, Murphy WM, Hautmann SH (2005). Bladder tumor markers beyond cytology: International Consensus Panel on bladder tumor markers.. Urology.

[7] Schmitz-Dräger BJ, Shariat SF, Droller M, Lokeshwar VB, Lotan Y, Khoury S (2012). Molecular markers for bladder cancer screening, early diagnosis, and surveillance.. ICUD Bladder Tumors. 2012 ed.

[8] Ambrosini G, Adida C, Altieri DC (1997). A novel anti-apoptosis gene, survivin, expressed in cancer and lymphoma.. Nat Med.

[9] Altieri DC (2008). Survivin, cancer networks and pathway-directed drug discovery.. Nat Rev Cancer.

[10] Verdecia MA, Huang H, Dutil E, Kaiser DA, Hunter T (2000). Structure of the human anti-apoptotic protein survivin reveals a dimeric arrangement.. Nat Struct Biol.

[11] Chantalat L, Skoufias DA, Kleman JP, Jung B, Dideberg O (2000). Crystal structure of human survivin reveals a bow tie-shaped dimer with two unusual alpha-helical extensions.. Mol Cell.

[12] Jeyaprakash AA, Klein UR, Lindner D, Ebert J, Nigg EA (2007). Structure of a Survivin-Borealin-INCENP core complex reveals how chromosomal passengers travel together.. Cell.

[13] Sun C, Nettesheim D, Liu Z, Olejniczak ET (2005). Solution structure of human survivin and its binding interface with Smac/Diablo.. Biochemistry.

[14] Wang F, Dai J, Daum JR, Niedzialkowska E, Banerjee B (2010). Histone H3 Thr-3 phosphorylation by Haspin positions Aurora B at centromeres in mitosis.. Science.

[15] Kelly AE, Ghenoiu C, Xue JZ, Zierhut C, Kimura H (2010). Survivin reads phosphorylated histone H3 threonine 3 to activate the mitotic kinase Aurora B.. Science.

[16] Yamagishi Y, Honda T, Tanno Y, Watanabe Y (2010). Two histone marks establish the inner centromere and chromosome bi-orientation.. Science.

[17] Mehrotra S, Languino LR, Raskett CM, Mercurio AM, Dohi T (2010). IAP regulation of metastasis.. Cancer Cell.

[18] Tran J, Master Z, Yu JL, Rak J, Dumont DJ (2002). A role for survivin in chemoresistance of endothelial cells mediated by VEGF.. Proc Natl Acad Sci U S A.

[19] Caldas H, Fangusaro JR, Boue DR, Holloway MP, Altura RA (2007). Dissecting the role of endothelial SURVIVIN DeltaEx3 in angiogenesis.. Blood.

[20] Hanahan D, Weinberg RA (2000). The hallmarks of cancer.. Cell.

[21] Shariat SF, Karakiewicz PI, Godoy G, Karam JA, Ashfaq R (2009). Survivin as a prognostic marker for urothelial carcinoma of the bladder: a multicenter external validation study.. Clin Cancer Res.

[22] Sarela AI, Macadam RC, Farmery SM, Markham AF, Guillou PJ (2000). Expression of the antiapoptosis gene, survivin, predicts death from recurrent colorectal carcinoma.. Gut.

[23] Adida C, Haioun C, Gaulard P, Lepage E, Morel P (2000). Prognostic significance of survivin expression in diffuse large B-cell lymphomas.. Blood.

[24] Kren L, Brazdil J, Hermanova M, Goncharuk VN, Kallakury BV (2004). Prognostic significance of anti-apoptosis proteins survivin and bcl-2 in non-small cell lung carcinomas: a clinicopathologic study of 102 cases.. Appl Immunohistochem Mol Morphol.

[25] Takai N, Miyazaki T, Nishida M, Nasu K, Miyakawa I (2002). Survivin expression correlates with clinical stage, histological grade, invasive behavior and survival rate in endometrial carcinoma.. Cancer letters.

[26] Altieri DC (2001). The molecular basis and potential role of survivin in cancer diagnosis and therapy.. Trends Mol Med.

[27] Swana HS, Grossman D, Anthony JN, Weiss RM, Altieri DC (1999). Tumor content of the antiapoptosis molecule survivin and recurrence of bladder cancer.. N Engl J Med.

[28] Smith SD, Wheeler MA, Plescia J, Colberg JW, Weiss RM (2001). Urine detection of survivin and diagnosis of bladder cancer.. JAMA.

[29] Shariat SF, Casella R, Khoddami SM, Hernandez G, Sulser T (2004). Urine detection of survivin is a sensitive marker for the noninvasive diagnosis of bladder cancer.. J Urol.

[30] Wang H, Xi X, Kong X, Huang G, Ge G (2004). The expression and significance of survivin mRNA in urinary bladder carcinomas.. J Cancer Res Clin Oncol.

[31] Weikert S, Christoph F, Schrader M, Krause H, Miller K (2005). Quantitative analysis of survivin mRNA expression in urine and tumor tissue of bladder cancer patients and its potential relevance for disease detection and prognosis.. Int J Cancer.

[32] Moussa O, Abol-Enein H, Bissada NK, Keane T, Ghoneim MA (2006). Evaluation of survivin reverse transcriptase-polymerase chain reaction for noninvasive detection of bladder cancer.. J Urol.

[33] Kenney DM, Geschwindt RD, Kary MR, Linic JM, Sardesai NY (2007). Detection of newly diagnosed bladder cancer, bladder cancer recurrence and bladder cancer in patients with hematuria using quantitative RT-PCR of urinary survivin.. Tumour Biol.

[34] Horstmann M, Bontrup H, Hennenlotter J, Taeger D, Weber A (2010). Clinical experience with survivin as a biomarker for urothelial bladder cancer.. World J Urol.

[35] Nasterlack M, Feil G, Leng G, Pesch B, Huber S (2011). Bladder cancer screening with urine-based tumour markers - occupational medical experience.. Aktuelle Urol.

[36] Pesch B, Nasterlack M, Eberle F, Bonberg N, Taeger D (2011). The role of haematuria in bladder cancer screening among men with former occupational exposure to aromatic amines.. BJU Int.

[37] Huber S, Schwentner C, Taeger D, Pesch B, Nasterlack M (2012). NMP22 - prospective evaluation in a population at risk for bladder cancer: results from the UroScreen-Study.. BJU Int.

[38] Lotan Y, Shariat SF, Schmitz-Drager BJ, Sanchez-Carbayo M, Jankevicius F (2010). Considerations on implementing diagnostic markers into clinical decision making in bladder cancer.. Urol Oncol.

[39] Nasterlack M, Scheuermann B, Messerer P, Pallapies D, Zober A (2001). Harnblasenkrebs in einem Risikokollektiv: Klinische und epidemiologische Aspekte.. Symp Med.

[40] Sanchez-Carbayo M, Urrutia M, Gonzalez de Buitrago JM, Navajo JA (2001). Utility of serial urinary tumor markers to individualize intervals between cystoscopies in the monitoring of patients with bladder carcinoma.. Cancer.

[41] Ransohoff DF, Gourlay ML (2010). Sources of bias in specimens for research about molecular markers for cancer.. J Clin Oncol.

[42] Weber DG, Casjens S, Rozynek P, Lehnert M, Zilch-Schoneweis S (2010). Assessment of mRNA and microRNA Stabilization in Peripheral Human Blood for Multicenter Studies and Biobanks.. Biomark Insights.

[43] Rathert P, Roth S (1991). Urinzytologie. Praxis und Atlas.

